# ClpB enhances thermotolerance in *Campylobacter jejuni* through protein disaggregation independent of DnaK

**DOI:** 10.1128/spectrum.02293-24

**Published:** 2025-04-30

**Authors:** Jeong In Hur, Jinshil Kim, Sangryeol Ryu, Byeonghwa Jeon

**Affiliations:** 1 Department of Food and Animal Biotechnology, Research Institute of Agriculture and Life Sciences, Seoul National University 366282, Seoul, South Korea; 2 Department of Agricultural Biotechnology, Seoul National University 539783, Gwanak-gu, Seoul, South Korea; 3 Center for Food and Bioconvergence, Seoul National University 26725, Gwanak-gu, Seoul, South Korea; 4 Department of Food Science and Biotechnology, and Carbohydrate Bioproduct Research Center, Sejong University 543336, Gwangjin-gu, Seoul, South Korea; 5 Division of Environmental Health Sciences, School of Public Health, University of Minnesota 246500 , Minneapolis, Minnesota, USA; University of Mississippi, University, Mississippi, USA

**Keywords:** *Campylobacter*, thermotolerance, multilocus sequence typing (MLST), ClpB, protein disaggregation

## Abstract

*Campylobacter jejuni* is a leading cause of foodborne infections worldwide and primarily transmitted to humans through the consumption of contaminated poultry meat. To enhance *Campylobacter*-associated food safety, it is critical to understand how *C. jejuni* survives during the thermal processing of poultry products. In this study, we monitored the survival of 86 *C*. *jejuni* strains during heat treatment and observed that some strains exhibited elevated heat tolerance. Notably, multilocus sequence typing clonal complex (CC)-443 and CC-607 were dominant among heat-tolerant strains, while the CC-21 strains were mostly heat-sensitive, indicating phylogenetic association with thermotolerance. We also investigated the function of heat shock chaperones in the thermotolerance of *C. jejuni*. Among several knockout mutants of heat shock chaperones, a mutant lacking *clpB* exhibited significantly lower survival than the wild type under heat treatment. Moreover, we observed a significantly higher accumulation of protein aggregates in the absence of *ClpB*, demonstrating that ClpB functions as a disaggregase during heat exposure. Additionally, ClpB from the heat-tolerant CC-443 group possessed distinct amino acid substitutions in the functional nucleotide-binding domain compared to ClpB in other CC groups. Interestingly, despite the well-known interaction of these proteins in many other bacteria, a two-hybrid assay demonstrated that ClpB of *C. jejuni* does not bind to DnaK, suggesting that *C. jejuni* may have a distinct mechanism for protein disaggregation and stress tolerance. Our findings demonstrate that ClpB plays a crucial role in the thermotolerance of *C. jejuni* through unique protein disaggregation mechanisms during poultry processing.

**IMPORTANCE:**

This study unveils a distinctive mechanism of thermotolerance involving protein disaggregation in *Campylobacter jejuni*, a major foodborne pathogen. Understanding *C. jejuni*’s ability to withstand heat stress is crucial for comprehending the occurrence of *Campylobacter* infections resulting from the consumption of contaminated poultry meat. Our research elucidates the roles of heat shock proteins, particularly ClpB, in the thermotolerance of *C. jejuni*. These findings significantly contribute to our fundamental understanding of bacterial physiology related to stress tolerance, which has important implications for public health and food safety.

## INTRODUCTION

*Campylobacter jejuni* is a predominant bacterial cause of foodborne illnesses globally (1, 2) and primarily transmitted through contaminated poultry products (3, 4). Symptoms of infection in humans include diarrhea, abdominal pains, and fever, with potential severe complications, such as Guillain-Barré syndrome and Miller-Fisher syndrome (5–7). *C. jejuni* thrives at an optimum temperature of 42°C, which suggests its ability to adapt to the higher body temperature of poultry in comparison to mammals (2). However, *C. jejuni* is more sensitive to environmental stress than other foodborne pathogens and has fastidious growth requirements (8), such as a narrow temperature range (from 30°C to 45°C) and a microaerobic atmosphere containing carbon dioxide (9). The sensitivity of *C. jejuni* to environmental stress can be ascribed to the absence of certain stress-adaptive responses that are conserved in other major foodborne pathogens, such as *Salmonella* and *Escherichia coli*. For instance, *C. jejuni* lacks *rpoS*‐encoded sigma factor stationary phase responsive genes, the major cold‐shock protein CspA, and the alternative sigma factor RpoH that regulates the heat‐shock response in *E. coli* (10, 11).

Limited information is available regarding the role of heat shock proteins in the thermotolerance of *C. jejuni*. Previous studies have demonstrated that *C. jejuni* increases expression levels of heat shock proteins (e.g., ClpB, DnaK, and GroESL) in response to heat stress (12, 13). Recent transcriptomic and proteomic studies have focused on the expression of chaperones and the binding of regulators under heat stress (14, 15). Additionally, changes in the binding affinity of heat shock regulators HrcA and HspR in the promoter region of heat shock proteins after heat stress in *C. jejuni* have been observed (15). HspR and HrcA negatively regulate the expression of three heat shock proteins: DnaK, ClpB, and GroESL (16–18). While many bacteria survive heat stress by utilizing heat shock proteins, ClpB is particularly well known for its contribution to bacterial survival in cooperation with DnaK by disaggregating protein aggregates formed under stress conditions (19, 20). Despite this knowledge, the specific role of heat shock proteins in *C. jejuni* remains unclear. Elucidating the heat shock defense mechanisms of *C. jejuni* in coping with heat stress is crucial for understanding foodborne infections resulting from the consumption of contaminated poultry products. This understanding could potentially lead to improved strategies for controlling *C. jejuni* contamination during food processing and ultimately enhance food safety.

The molecular mechanisms underlying the adaptive response of *C. jejuni* to heat stress, particularly during poultry processing steps, remain poorly understood. Specifically, during poultry processing, chickens may be subjected to scalding treatments at temperatures around 50–60°C to facilitate feather removal. This scalding process is critical, as it can significantly impact the microbial load of pathogens, such as *C. jejuni*, on chicken skin. Despite these treatments, *C. jejuni* is often resilient and can survive, leading to continued food safety concerns (21). Meanwhile, understanding the genetic diversity among different lineages of *C. jejuni*, particularly clonal complexes (CCs), is crucial for assessing their ecological and epidemiological implications. Variations in heat tolerance among *C. jejuni* lineages, especially those identified through multilocus sequence typing (MLST), could significantly influence their survival and success during food processing and under environmental stress. However, to date, there has been no established correlation between specific CCs and thermotolerance in the literature. This absence of information indicates that the relationship between strain lineage and stress resistance remains complex and not yet fully understood. A previous study has highlighted the variability among *C. jejuni* lineages without correlating these strains with heat tolerance (22). Similarly, findings from Oh et al*.* (23) characterized the stress tolerance of *C. jejuni* strains isolated from retail raw chicken, reinforcing the notion that comprehensive analyses linking clonal complexes to thermotolerance are lacking. Furthermore, our latest studies explored the phylogenetic associations and genetic factors related to cold stress tolerance but did not establish connections to thermotolerance (24, 25). Therefore, our investigation focuses on elucidating the mechanisms that underlie the divergent heat tolerance observed among *C. jejuni* lineages, contributing to food safety and public health.

In this study, we reveal a significant correlation between bacterial phylogeny and thermotolerance: MLST CC-443 was predominant among heat-tolerant strains, whereas CC-21 was prevalent in heat-sensitive strains. This phylogenetic association suggests the existence of specific genetic lineages of *C. jejuni* with enhanced thermotolerance. Furthermore, we demonstrate that the heat shock protein ClpB plays a crucial role in *C. jejuni*’s heat tolerance by disaggregating heat-denatured proteins under thermal stress conditions.

## MATERIALS AND METHODS

### Bacterial strains and culture conditions

Eighty-six *C. jejuni* strains, which were isolated from retail raw chicken in our previous study (26), were used to monitor thermotolerance. *C. jejuni* NCTC 11168, the first genome-sequenced strain in *Campylobacte*r (27), was used as a wild type in this study. *C. jejuni* strains were routinely grown on Mueller-Hinton media (Oxoid, Hampshire, UK) at 42°C under microaerobic conditions (5% O_2_, 10% CO_2_, and 85% N_2_) generated by Anoxomat (Mart Microbiology BV, Lichtenvoorde, Netherlands). *E. coli* was grown at 37°C aerobically in Luria-Bertani media (BD Difco, MD, USA) supplemented with carbenicillin (Cb, 100 µg/mL) or kanamycin (Kan, 50 µg/mL) when required.

### Construction of *C. jejuni* mutants and complemented strains

Construction of *C. jejuni* NCTC11168 *clpB*, *dnaK*, and *groESL* deletion mutants, as well as their respective complemented strains, has been described previously (28). For the construction of *hrcA* and *hspR* mutants and complemented strains*,* we selected the *C. jejuni* NCTC 11168 as a reference strain as it is commonly used in many laboratories (27). Suicide plasmids carrying *hrcA* and *hspR* were constructed as described previously (29). Briefly, each gene and its flanking region were amplified from *C. jejuni* NCTC 11168 by PCR with GXL polymerase (TaKaRa, Tokyo, Japan) using the primers listed in Table S2. After digestion with *Sal*I and *Bam*HI, the PCR products were each ligated to pUC19 that had been treated with the same enzymes. The pUC19 plasmid containing each gene was amplified by PCR from inside the gene with inverse primers using the same polymerase and ligated with a kanamycin resistance cassette amplified from pMW10 using the Kan-F and Kan-R primers (Table S2). The suicide vectors were commercially sequenced by Bionics (Seoul, Republic of Korea). These plasmids were used as suicide vectors, and each vector was introduced into *C. jejuni* NCTC 11168 by electroporation. Mutants were screened by growing on MH agar containing kanamycin (50 µg/mL), and the mutations were confirmed by PCR and sequencing.

Complemented strains were constructed by chromosomal integration as previously described (30). Briefly, DNA fragments containing an intact copy of *hrcA* and *hspR* were amplified with primer pairs (Table S2) and ligated to the *Not*I site of a pUC19 derivative carrying an rRNA gene cluster, pFMBcomCM. pFMBcomCM facilitates the chromosomal integration of the complemented genes. The expression of these genes inserted into pFMBcomCM is driven by the native rRNA promoter, allowing for the proper regulation of gene expression in the context of the *C. jejuni* cellular environment. Plasmids carrying the genes were sequenced by Bionics (Seoul, Republic of Korea) and used as complementation vectors. The complementation vectors were introduced into Δ*hrcA* and Δ*hspR* mutants by electroporation. The complemented strains were selected by growing on MH agar plates supplemented with kanamycin (50 µg/mL) or chloramphenicol (12.5 µg/mL). The complementation of each gene was confirmed by PCR and sequencing.

### Thermotolerance test

An overnight culture grown on MH agar was resuspended in MH broth to an optical density of 600 nm (OD_600_) of 0.1 (ca, 10^9^ CFU/mL). The bacterial suspension was transferred to 0.2 mL 8-Strip PCR SnapStrip Tubes, Flat Cap (SSIbio, CA, USA) in 100 µL aliquots. The PCR tubes were sealed and incubated at 50°C for 60 min, and samples were taken at 10 or 20 min intervals for serial dilution and bacterial counting. The detection limit of the assay was 200 CFU/mL.

### Determination of delta (*D*)-value

*D*-values, the time required to kill 90% of the organism during heat treatment, were determined by the linear regression technique (31, 32). Multiple linear regression models with *R*^2^ values were higher than 0.85 for all cases. Thermal inactivation curves were determined by plotting populations of *C. jejuni* on a logarithmic scale as a function of heating time at 50°C. *D*-values were calculated from the slope of a simple regression line for each thermal inactivation curve at 50°C.

### Thermotolerance tests under chicken scalding conditions

*C. jejuni*-spiked chicken skin samples were prepared as previously described (22, 24). Pieces of raw chicken skin (≈1 cm^2^/piece) were prepared from retail raw chicken skin by cutting with a sterile scissor. Raw chicken skin pieces were dipped in 70% ethanol overnight at 4°C to eliminate bacterial contaminants. The remaining contaminants were sterilized by exposing the chicken skin pieces to ultraviolet light on each side for 30 min. The sterilized-dried chicken skin pieces were placed into 24-well plates. *C. jejuni* suspensions were prepared by diluting an overnight culture to an OD_600_ of 0.1 with Dulbecco’s phosphate-buffered saline without calcium and magnesium (DPBS) (GenDEPOT, TX, USA). An aliquot of 10 µL of culture (ca, 10^7^ CFU) was inoculated to the surface of chicken skin to obtain an initial population of approximately 10^6^ CFU/mL after dipping in 0.1% buffered peptone water (BPW) (KisanBio, Seoul, Republic of Korea). Negative controls were prepared by inoculating 10 µL of DPBS on chicken skin. Then, all inoculated skin samples were left at room temperature for 30 min to allow bacterial cells to adhere to the skin surfaces.

To determine the impact of thermotolerance on poultry processing interventions, we mimicked the conditions of chicken scalding treatment as described previously with slight modifications (21, 33). A 50 mL falcon tube containing 30 mL of 0.1% BPW was heated at 50°C in the water bath for 30 min before the test. Then, chicken skin pieces spiked with *C. jejuni* were individually dipped into each pre-warmed falcon tube containing 0.1% BPW and incubated at 50°C for 5 min. Every 1 min for 5 min, the chicken skin was taken from the falcon tube with a sterile tweezer, transferred to 15 mL tubes containing 1 mL of 0.1% BPW, and vigorously vortexed for 2 min. Supernatants were collected for serial dilution and bacterial counting. The detection limit of the assay was 100 CFU/mL.

### Bacterial adenylate cyclase two-hybrid system assay

The bacterial adenylate cyclase two-hybrid (BACTH) system was used to examine the protein interaction between ClpB and DnaK. Protein interaction assays were performed following the previously described protocol (34). Briefly, for the BACTH constructs, ClpB and DnaK were amplified from *C. jejuni* NCTC 11168 by PCR with GXL polymerase (TaKaRa, Tokyo, Japan) using the primers in Table S1. After digestion with *Xba*I and *Kpn*I, the PCR products of ClpB and DnaK were each ligated to pUT18C and pKT25, which had been treated with the same enzymes. Each pUT18C and pKT25 plasmid was co-transformed into chemically competent *E. coli* BTH101 (*cya* mutant) and plated in LB-X-Gal plates. BACTH screening LB-X-Gal plates include LB agar supplemented with carbenicillin (Cb, 100 µg/mL) and kanamycin (Kan, 50 µg/mL) with 20 µg/mL 5-bromo-4-chloro-3-indolyl-β-D-galactopyranoside (X-Gal) (Duchefa Biochemie, Haarlem, The Netherlands). For positive control, ClpB and DnaK of *E. coli* MG1655 were used in both pUT18C and pKT25 backgrounds, as they have previously been shown to interact (19, 35) (Table S1). Negative controls were constructed using empty vectors of pUT18C and pKT25. Colonies were individually picked and grown overnight at 37°C in LB supplemented with Cb or Kan. This cell suspension was used as inoculum for all subsequent screening and growth experiments.

The β-galactosidase levels were determined by two methods. To culture cells for β-galactosidase activity measurement, cells grown overnight were sub-cultured in LB media supplemented with Cb or Kan, and 0.5 mM isopropyl β-D-1-thiogalactopyranoside (IPTG, Sigma-Aldrich, MO, USA) was added to induce protein expression. For plate assays, 10 µL of sub-cultured cells was spotted on LB-X-Gal plates, and plates were incubated at 30°C for 48 h under aerobic conditions. For quantitative measurement, β-galactosidase activity was determined using a liquid assay (36). Subcultures were centrifuged, and pellets were resuspended with Z-buffer (60 mM Na_2_HPO_4_·7H_2_O, 60 mM NaH_2_PO_4_·H_2_O, 10 mM KCl, 1 mM MgSO_4_, 50 mM β-mercaptoethanol, adjusted to pH 7.0) to an OD_600_ of 0.7 (blank against Z-buffer). To permeabilize the bacterial cells, sodium dodecyl sulfate and chloroform were added, followed by a light vortex for 10 s, and incubated at room temperature for 10 min. Then, o-nitrophenol-β-galactoside (ONPG; 4 mg/mL) was mixed with gentle inverting. Finally, the reaction was stopped by raising the pH of the solution with 1 M Na_2_CO_3_ precisely after 30 min of incubation time at 37°C. The OD_420_ and OD_550_ were determined and normalized to the cell density in the washed cell suspension (OD_600_), the volume of culture used in milliliters, and the time in minutes of incubation prior to halting the reaction. The β-galactosidase activity was recorded in Miller units.

### Quantification of protein aggregates

*C. jejuni* cultures were taken after 0, 10, and 20 min exposure to 50°C, washed twice with ice-cold DPBS (GenDEPOT, TX, USA), and centrifuged at 10,000 ×*g* for 5 min. Following the manufacturer’s instructions, samples were stained with PROTEOSTAT aggresome detection reagent (Enzo Life Sciences, Inc., NY, USA). The PROTEOSTAT dye precisely intercalates into the cross-beta spine of aggregated and misfolded proteins, which hinders the dye’s rotation and leads to strong fluorescence. Briefly, pellets were stained in the dark with PROTEOSTAT by incubating at room temperature for 15 min. Samples were placed onto a 96-well plate (black opaque; Corning, NY, USA), and fluorescence was measured with the SpectraMax i3 platform (Molecular Devices, CA, USA) at 500  nm excitation and 600  nm emission wavelengths. The relative fluorescence units (RFU) were normalized to CFU/mL of the sample and reported in RFU/CFU.

### Confocal fluorescence microscopy

Overnight cultures of *C. jejuni* grown on MH agar were suspended in MH broth to an OD_600_ of 0.1. The bacterial suspension was transferred to a disposable culture tube (Kimble, NJ, USA) and incubated at 42°C with shaking (200  rpm) for 6 h. Bacterial cultures were suspended in MH broth to an OD_600_ of 0.1 and transferred to a PCR tube in 100 µL aliquots. The PCR tubes were heat treated at 50°C for 20 min.

For staining and fixing steps, samples incubated at 50°C were taken after 0, 10, and 20 min, washed twice with ice-cold DPBS, and centrifuged at 10,000 ×*g* for 5 min. Pellets were stained with SYTO 9 Green Fluorescent Nucleic Acid Stain (Invitrogen, MA, USA) and PROTEOSTAT dye, incubating for 20 and 15 min, respectively, at room temperature without light. Then, pellets were washed with ice-cold DPBS and fixed in 4% paraformaldehyde in DPBS at room temperature for 20 min. After fixation, bacterial cells were washed twice with ice-cold DPBS to observe fluorescence.

Slide glass (Paul Marienfeld GmbH & Co. KG, Laud-Königshofen, Germany) with a thickness of 1 mm was coated with 0.01% (w/v) poly-L-lysine solution (Sigma-Aldrich, MO, USA) to improve cell attachment. Then, each sample (5 µL) was dropped onto a poly-L-lysine-coated slide glass. Samples covered with a cover glass were examined on a confocal laser scanning microscope SP8 X (Leica, Wetzlar, Germany) using appropriate filters with fixed excitation/emission wavelengths at 488  nm/500–600 nm for SYTO 9 and excitation/emission wavelengths at 488 nm/600–700 nm for the PROTEOSTAT dye.

### Cross-sectional transmission electron microscopy

Bactericidal protein aggregation can be observed by transmission electron microscopy (TEM) of cross-sections of resin-embedded bacteria (37). Samples incubated at 50°C were taken after 0, 10, and 20 min, washed twice with ice-cold DPBS, and centrifuged at 10,000 ×*g* for 5 min. Pellets were fixed at 4°C overnight with Karnovsky’s fixative consisting of 3% glutaraldehyde and 2% paraformaldehyde in 0.05 M sodium cacodylate buffer (pH 7.2). After primary fixation, each sample was washed thrice with 0.05 M sodium cacodylate buffer at 4°C for 5 min. Each sample was post-fixed for 1 h at room temperature with 0.05 M sodium cacodylate buffer containing 1% osmium tetroxide. The fixed sample was rinsed three times with distilled water at 4°C for 5 min and stained overnight with 0.5% uranyl acetate in distilled water at 4°C. The stained samples were dehydrated at room temperature in an ethanol gradient of 30, 50, 70, and 90% for 20 min and finally repeated three times at 100%, each step with slow rotation. Finally, cells were infiltrated with slow rotation using a 1:1 mix of 100% ethanol and Spurr’s resin for 2 h, following 2:1 of 100% ethanol and Spurr’s resin, and subsequently left in 100% Spurr’s resin overnight. For final infiltration, samples were re-immersed in 100% fresh Spurr’s resin for 2 h under slow rotation and polymerized for 24 h at 70°C. The polymerized specimen block was sectioned with a 5 nm thickness using an Ultramicrotome EM UC7 (Leica, Wetzlar, Germany). The cross-sectional images were observed using a transmission electron microscope JEM1010 (JEOL, Tokyo, Japan) at an accelerating voltage of 80 kV.

### Sequence alignment and phylogenetic analysis

The phylogenetic tree was visualized via iTOL v6 based on amino acid sequence alignments of ClpB homologs generated by CLC Main Workbench v7.7.1.

### Statistical analysis

Student’s *t*-test was performed for comparative analysis between the two groups. A *χ* test was conducted to compare the proportion of the two groups. GraphPad Prism v8.0.1 (GraphPad Software, Inc., CA, USA) was used for statistical analysis.

## RESULTS

### *C. jejuni* exhibits diverse thermotolerance profiles with phylogenetic association

**Fig 1:**
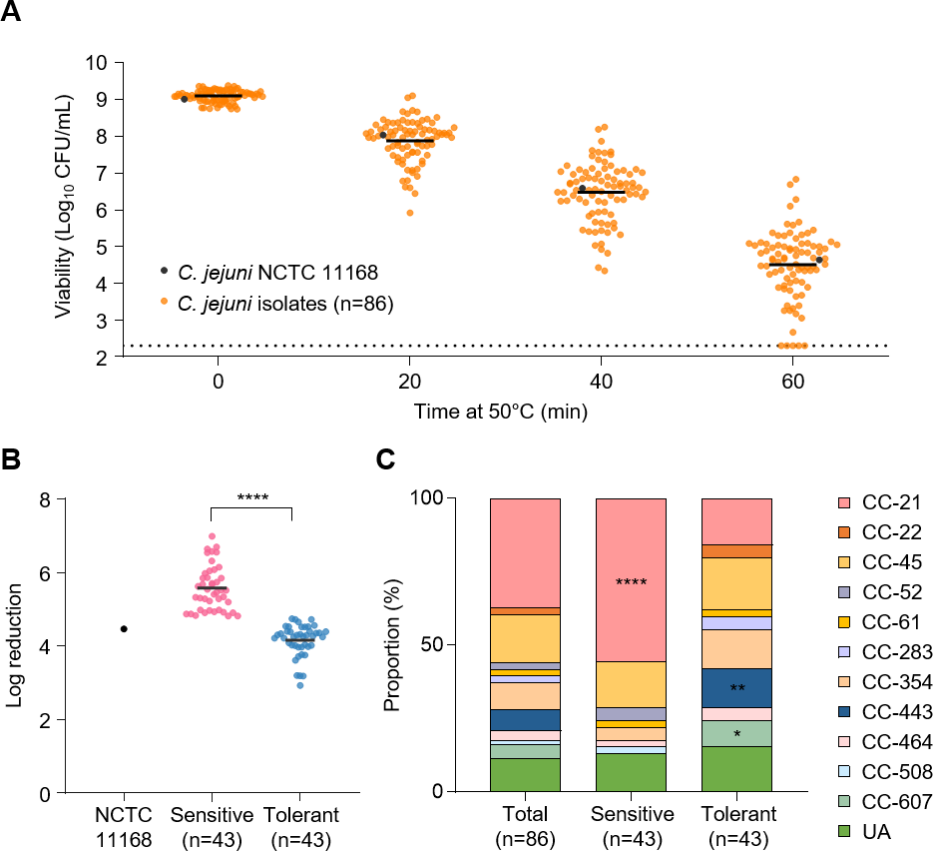
Differential survival of 87 *C*. *jejuni* strains under heat stress and variations in the distribution of multilocus sequence typing (MLST) clonal complexes (CCs) between heat-sensitive and heat-tolerant strains. (A) Viable counts of 87 *C*. *jejuni* strains were measured at 0, 20, 40, and 60 min of exposure to 50°C in Mueller-Hinton broth. The experiment was repeated three times, and each dot represents the average of three replicates. The dotted line indicates the detection limit (200 CFU/mL). (B) Log reduction values of heat-sensitive and heat-tolerant strains after 60 min of heat treatment. In panels A and B, colored circles represent *C. jejuni* strains as follows: orange, *C. jejuni* isolates (*n* = 86); pink, heat-sensitive strains (*n* = 43); blue, heat-tolerant strains (*n* = 43); and black, reference strain (*C. jejuni* NCTC 11168). The experiment was performed in triplicate with consistent results. Black lines indicate mean values. (C) Comparison of MLST CC proportions between heat-sensitive (*n* = 43) and heat-tolerant strains (*n* = 43). Student’s *t*-test was used to compare viabilities between heat-sensitive and heat-tolerant strains. A *χ* test was conducted to compare the proportions of CCs. Significance levels are denoted as follows: *, *P* < 0.05; **, *P* < 0.01; ****, *P* < 0.0001. CC, clonal complex; UA, unassigned to any defined CC.

*C. jejuni* grows at temperatures ranging from 37 to 45°C, with an optimum at 42°C, and is considered thermotolerant (2). While most pathogenic *Campylobacter* species are thermophilic, they are typically sensitive to heat treatments above 50°C, which is a temperature range for pasteurization and cooking. Despite heat treatments during processing, such as the scalding of chicken carcasses, *C. jejuni* is still frequently found in retail poultry meat (21, 38). To understand the mechanisms underlying the survival of *C. jejuni* under heat stress, we measured the survival of 86 *C*. *jejuni* strains at 50°C, which were isolated from retail raw chicken in our previous study (26). *C. jejuni* NCTC 11168 was selected as a reference strain, as it is one of the common *C. jejuni* strains used by many laboratories (27). The 86 strains that were tested exhibited various survival patterns in their viability at 50°C for 60 min (Fig. 1A). For a comparative analysis of their thermotolerance, based on the log reduction values after heat treatment for 60 min, 86 strains were divided into two groups of equal size (*n* = 43). The group with a higher log reduction value was classified as heat-sensitive, while the other group was classified as heat-tolerant (Fig. 1B). The log reduction value of the dividing point was approximately 4.72 at 60 min. Heat-sensitive strains (*n* = 43) showed significantly higher log reduction values (*P* < 0.0001) compared to heat-tolerant strains (*n* = 43).

Among the heat-sensitive strains, those belonging to MLST CC-21 accounted for 55.6% of the population, indicating a prevalence of these strains (*P* < 0.001) (Fig. 1C). Strains belonging to CC-45, CC-61, CC-354, and UA present in the heat-sensitive population were not found to be statistically dominant among the heat-sensitive strains when compared to the heat-tolerant strains (Fig. 1C). In contrast, a significant proportion of heat-tolerant strains belonged to CC-443 and CC-607 (13.3 and 8.9%, respectively) and exhibited a high proportion (*P* < 0.01and *P* < 0.05, respectively) (Fig. 1C). These results indicate that *C. jejuni* exhibits diverse survival patterns under heat stress, and thermotolerance is associated with its phylogeny.

### ClpB plays a critical role in *C. jejuni* survival under scalding conditions

**Fig 2:**
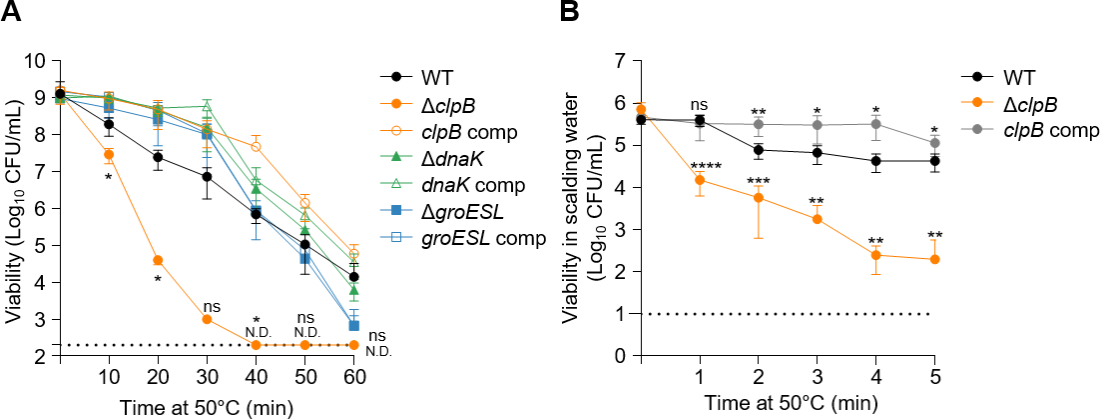
Contribution of *clpB* to thermotolerance in *C. jejuni*. (A) Survival of *C. jejuni* strains was measured during 60 min of exposure to 50°C. The dotted line indicates the detection limit (200 CFU/mL). (B) Viability of *C. jejuni* strains measured after exposure to scalding water at 50°C for 5 min. The dotted line indicates the detection limit (100 CFU/mL). Data shown are representative of three independent experiments with similar results. Error bars represent standard errors of the means. Statistical analysis was performed using Student’s *t*-test to compare viabilities between WT and indicated strains at each sampling time point. Significance levels are denoted as follows: *, *P* < 0.05; **, *P* < 0.01; ***, *P* < 0.001; ****, *P* < 0.0001; ns, non-significant; N.D., not detected; WT, *C. jejuni* NCTC 11168 wild type; Δ*clpB*, Δ*clpB* mutant; *clpB* comp, *clpB*-complemented strain; Δ*dnaK*, Δ*dnaK* mutant; *dnaK* comp, *dnaK*-complemented strain; Δ*groESL*, Δ*groESL* mutant; *groESL* comp, *groESL*-complemented strain.

Bacterial cells respond to exposure to temperatures above optimal growth through synthesizing heat shock proteins (11). We sought to elucidate the role of heat shock proteins in the thermotolerance of *C. jejuni* during poultry processing steps that involve heat treatment. The wild type background used in our experiments was *C. jejuni* NCTC 11168, which belongs to CC-21. First, we tested knockout mutants defective in heat shock chaperone genes, including *clpB*, *dnaK,* and *groESL*, which are repressed by two heat shock response regulators, HrcA and HspR, under normal conditions (14, 16). We evaluated the survival of heat shock chaperone mutants at 50°C (Fig. 2A). A Δ*clpB* knockout mutant showed a rapid decrease in its viability at 50°C and failed to recover after 40 min of heat treatment (Fig. 2A). Meanwhile, the viability of other mutants, including a Δ*dnaK* knockout mutant, remained comparable to that of WT during heat treatment (Fig. 2A). Next, we determined delta (D)-values with survival data from the heat treatment experiment (Fig. 2A) using the log-linear model (Fig. S1). The D-value refers to the time required at a specific temperature to reduce the viable population of an organism by 90%, or one log cycle. This parameter is crucial for understanding the thermal inactivation kinetics of *C. jejuni* during heat treatment. When D-values were compared, the Δ*clpB* mutant showed significantly different standard errors of the mean (*P* < 0.0001) compared to WT (Table 1). The D-value markedly decreased to 4.79 min in the Δ*clpB* mutant, while WT showed 12.45 min at 50°C, and the *clpB*-complemented strain restored its D-value up to 13.46 min (Table 1). These results indicate that ClpB plays a critical role in the thermotolerance of *C. jejuni*.

**TABLE 1:** Delta (*D*) value of *C. jejuni* strains at 50°C based on log-linear regression

Initial population	WT		Δ*clpB*		*clpB* comp	
(CFU/mL)	*D*-value (min)	R^2^	*D*-value (min)	R^2^	*D*-value (min)	*R* ^2^
1 × 10^9^	12.45	0.96	4.79 ± 0.01^*a*^	0.9877	13.46 ± 0.01	0.8576

^*a*^ Standard error of the mean differs significantly compared to WT (*P* < 0.0001). Student’s *t*-test was performed for statistical analysis between *D* values. The *D* values in min were averaged from three independent trials. WT, *C. jejuni* NCTC 11168 wild type; Δ*clpB*, Δ*clpB* mutant; *clpB* comp, *clpB*-complemented strain.

Previous reports have shown that the *hrcA·grpE·dnaK* operon and the *groESL* operon are both cooperatively regulated by HspR and HrcA, while *clpB* is regulated solely by HspR (14, 16) (Fig. S2A). To further investigate the role of these heat shock regulators, we assessed the survival of knockout mutants of the heat shock regulator genes *hrcA* and *hspR*. During heat treatment, the Δ*hspR* mutant exhibited significantly enhanced survival compared to the WT strain (Fig. S2B). The elevated survival of the Δ*hspR* mutant during heat treatment can suggest a potential derepression of ClpB, but further empirical validation regarding the interaction and regulation of these heat shock proteins seems necessary. Genetic complementation of the Δ*hspR* mutant with an intact copy of *hspR* fully restored thermotolerance to WT levels (Fig. S2B). These results corroborate previous studies (14, 16) and suggest that ClpB significantly contributes to the thermotolerance of *C. jejuni*.

To evaluate the impact of ClpB on *C. jejuni* survival under conditions mimicking chicken scalding procedures, we assessed the viability of *C. jejuni* on chicken skin exposed to a scalding temperature of 50°C. The Δ*clpB* mutant exhibited a significant decrease in viability compared to the WT strain (Fig. 2B). While WT maintained its viability until the end of the experiment (5 min) with less than one log reduction (Fig. 2B), the Δ*clpB* mutant experienced a rapid decline in viability, showing approximately a 5-log reduction after 5 min (Fig. 2B). Notably, the genetic complementation of the Δ*clpB* mutant restored its survival under scalding conditions to WT levels (Fig. 2B). These findings strongly suggest that ClpB plays a crucial role in the survival of *C. jejuni* under heat stress conditions.

### *C. jejuni* ClpB does not bind with DnaK

**Fig 3:**
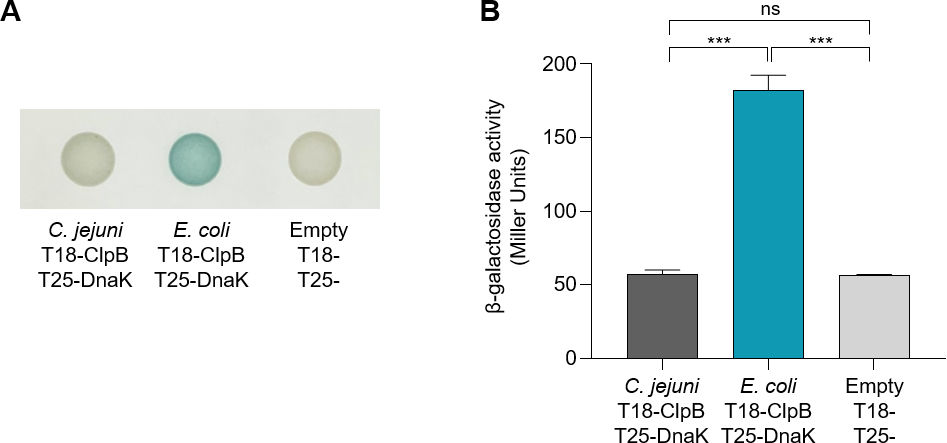
*C. jejuni* ClpB does not interact with DnaK. (A) Bacterial adenylate cyclase two-hybrid (BACTH) assays demonstrating heterotypic interactions between ClpB and DnaK of *C. jejuni*. Positive (T18-ClpB of *E. coli* versus T28-DnaK of *E. coli*) and negative controls (T18-empty vector versus T28-empty vector) are shown for comparison. (B) Quantification of *β*-galactosidase activity from the BACTH interactions shown in panel A, expressed in Miller units. The experiment was performed in triplicate. Error bars represent standard errors of the means. Statistical analysis was conducted using Student’s *t*-test. Significance levels are denoted as follows: ***, *P* < 0.001; ns, non-significant.

Previous studies in *E. coli* have demonstrated that the cooperation of DnaK with ClpB is crucial for protein disaggregation, involving direct binding and interaction between the two proteins (19, 35). Moreover, a rapid decrease in bacterial survival under heat treatment has been observed in an *E. coli* Δ*dnaK* mutant (39). In contrast, our results showed no decrease in the survival of the *C. jejuni* Δ*dnaK* mutant under heat treatment (Fig. 2A). To further assess protein-protein interactions, we performed BACTH assays using both X-gal indicator plates and liquid assays. These experiments revealed that DnaK and ClpB in *C. jejuni* do not bind to each other, whereas DnaK and ClpB in *E. coli* exhibited clear interaction (Fig. 3A and B). These findings suggest that ClpB contributes to the thermotolerance of *C. jejuni* under heat stress without direct protein binding to DnaK. This distinct mechanism in *C. jejuni*, differing from the well-characterized DnaK-ClpB interaction in *E. coli*, demonstrates the unique adaptations of *C. jejuni* to thermal stress.

### ClpB assists *C. jejuni* in mitigating protein aggregates caused by thermal stress

**Fig 4:**
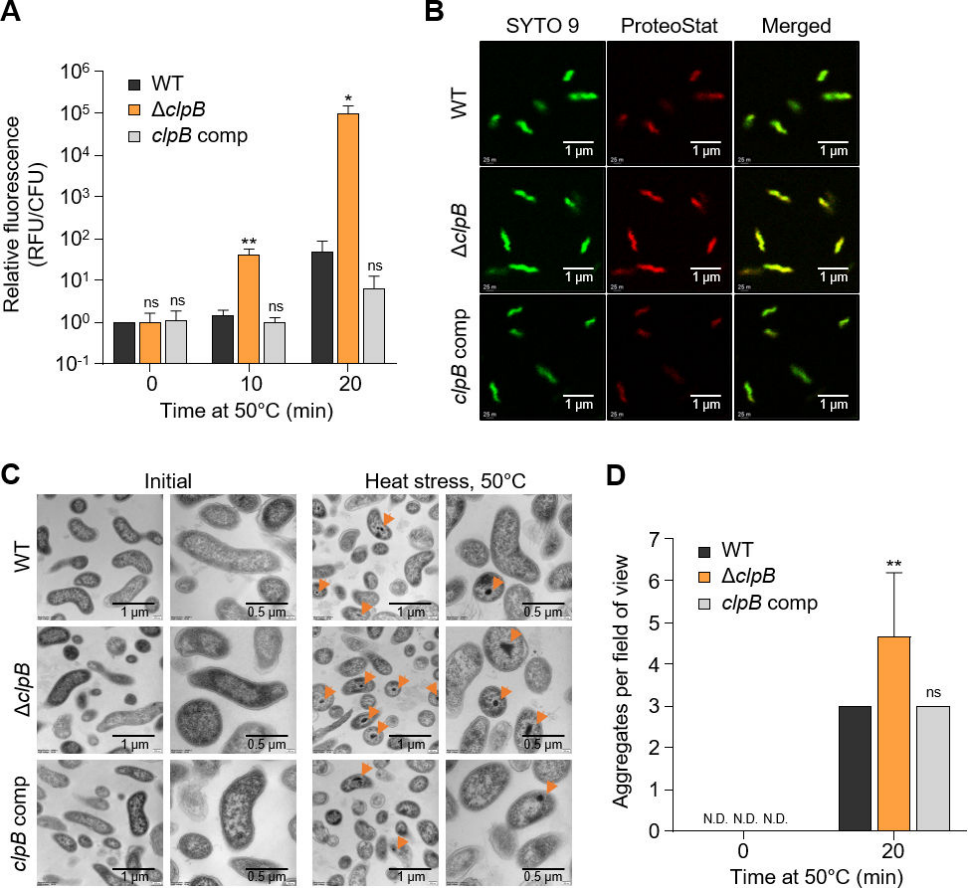
Protein disaggregation by ClpB of aggregates formed under heat stress in *C. jejuni*. (A) Comparison of aggregated protein levels during heat treatment at 50°C for 20 min. The fluorescence intensity of the WT strain before heat treatment (0 min) was set as 1. (B) Confocal microscopy images of protein aggregates using SYTO 9 dye (green) and PROTEOSTAT dye (red) in samples heat-treated for 20 min. Merged images of SYTO 9 and PROTEOSTAT dye staining are shown on the right. (C) Cross-sectional transmission electron microscopy (TEM) images of *C. jejuni* strains before (Initial) and after heat treatment for 20 min at 50°C (heat stress). Protein aggregates are indicated by orange arrows. (D) Aggregates per field of view obtained from cross-sectional TEM images of *C. jejuni* strains before (0 min) and after heat treatment at 50°C for 20 min. The data shown are representative of three independent experiments with similar results. Error bars represent standard errors of the means. Statistical analysis was performed using Student’s *t*-test. Significance levels are denoted as follows: *, *P* < 0.05; **, *P* < 0.01; ns, non-significant. WT, *C. jejuni* NCTC 11168 wild type; Δ*clpB*, Δ*clpB* mutant; *clpB* comp, *clpB*-complemented strain.

ClpB is a chaperone that mediates the transformation of aggregated proteins to their native state by disaggregating protein aggregates formed under stress conditions (19). In many bacterial species, ClpB is known to function cooperatively with DnaK as a disaggregase under heat stress (20, 35). However, our findings in *C. jejuni* reveal that DnaK does not play a significant role in survival during heat treatment, nor does it interact with ClpB (Fig. 2A and 3). To elucidate the role of ClpB in protein disaggregation in *C. jejuni*, we examined the formation of protein aggregates after heat treatment. We measured protein aggregates using the PROTEOSTAT dye, which binds to the quaternary protein structures found in misfolded and aggregated proteins, causing strong red fluorescence by inhibiting the dye’s rotation (40). Relative fluorescence levels increased in all samples after heat treatment, indicating the formation of protein aggregates due to heat exposure (Fig. 4A). Notably, the Δ*clpB* mutant showed significantly higher levels of protein aggregates compared to WT after 10 min and 20 min of heat treatment (*P* < 0.01 and *P* < 0.05, respectively) (Fig. 4A).

To further validate these findings, we employed two widely used techniques for observing protein aggregates: fluorescent dye staining and cross-sectional transmission electron microscopy (TEM) (37, 41). After 20 min exposure to heat treatment, the PROTEOSTAT dye was detected at higher levels in the Δ*clpB* mutant compared to both the WT and *clpB*-complemented strains (Fig. 4B), indicating increased protein aggregation in the Δ*clpB* mutant. Moreover, cross-sectional TEM visualization of the cells revealed more extensive formation of protein aggregates in the Δ*clpB* mutant compared to the WT and *clpB*-complemented strains after 20 min of heat treatment (*P* < 0.01) (Fig. 4C and D). Collectively, these results provide strong evidence that ClpB plays a critical role in the thermotolerance of *C. jejuni* by mitigating the formation of protein aggregates during thermal stress.

### Distinct polymorphisms in ClpB in the CC-443 group

**Fig 5:**
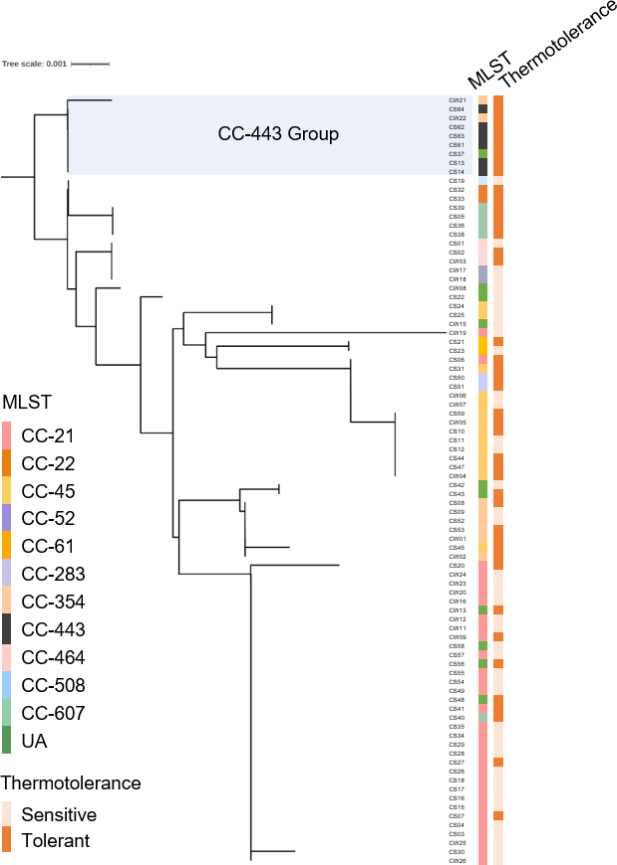
Analysis of ClpB amino acid sequences from *C. jejuni* isolates. (A) Phylogenetic tree based on ClpB amino acid sequences from 86 *C*. *jejuni* isolates. CC, clonal complex; UA, unassigned to any defined CC.

Our observations of differential thermotolerance among various *C. jejuni* strains (Fig. 1) and the significant role of ClpB in this process (Fig. 2 and 4) led us to hypothesize that thermotolerance may be associated with polymorphisms in the ClpB protein sequence. To investigate this, we conducted a phylogenetic analysis using ClpB amino acid sequences from 86 *C*. *jejuni* isolates (Fig. 5). The amino acid sequence of ClpB in strains CW21 (CC-354), CW22 (CC-354), and CS37 (UA) is identical to the ClpB sequence found in all six CC-443 strains (CS13, CS14, CS61, CS62, CS63, and CS64). Consequently, we have designated a total of nine strains sharing this identical sequence as part of the CC-443 group. The ClpB of the CC-443 group exhibited 99.18% identity (850/857 amino acids) with no gaps compared to *C. jejuni* NCTC 11168, which shares an identical sequence with the CC-21 group (Fig. 5; Fig. S3). Based on the alignment of the ClpB amino acid sequences, we predicted the domain organization of *clpB* homologs from the CC-443 group and *C. jejuni* NCTC 11168 (Fig. S3). As the domains of *C. jejuni* ClpB have not been previously characterized, we used the domains of *E. coli* ClpB, which consists of the same length (857 amino acids), as a reference for domain prediction. Seven amino acid substitutions were identified in the CC-443 group compared to *C. jejuni* NCTC 11168: A20V, S44N, T45A, M150I, A474T, A596T, and N726S (Fig. S3).

Notably, ClpB from the CC-443 group exhibited distinct amino acid substitutions in the N-terminal domain (NTD) and the second nucleotide-binding domain (NBD-2). Specifically, we observed changes from alanine (A) to valine (V) in the NTD and to threonine (T) in NBD-2 compared to ClpB in other CC groups (Fig. S3). The NTD of ClpB plays a crucial role in substrate binding and disaggregation activity, although it is dispensable for oligomerization and ATPase activity (42). NBD-1 and NBD-2 exhibit distinct properties, with NBD-2 showing higher affinity for nucleotides and greater ATPase activity (43, 44). These results suggest that although direct data demonstrating their impact on protein folding compared to variants are lacking, these unique substitutions in the NTD and NBD-2 of ClpB may correlate with the differential heat stress tolerance capabilities observed in *C. jejuni* strains (Fig. 1A).

## DISCUSSION

In this study, we sought to elucidate the survival mechanisms of *C. jejuni* during heat stress by first investigating the phylogenetic relationship between different survival rates among 86 *C*. *jejuni* strains isolated from retail raw chicken. Our results demonstrate that *C. jejuni* exhibits strain-dependent variation in survival ability under heat treatment (Fig. 1A). Comparative analysis of MLST and thermotolerance revealed a significant association between *C. jejuni*’s phylogenetic background and its thermotolerance (Fig. 1C). Specifically, strains belonging to CC-443 were represented in significantly higher proportion among heat-tolerant strains, whereas CC-21 strains generally displayed sensitivity to heat stress (Fig. 1C). These findings align with our previous studies, which demonstrated that CC-443 strains are also tolerant to aerobic and cold stresses (24–26), suggesting a broader stress resistance phenotype for this clonal complex. Surprisingly, CC-443 strains of *C. jejuni* show a significant correlation between human and poultry sources. According to the PubMLST database (www.pubMLST.org), among the 81 CC-443 isolates reported thus far, human stool (58.1%) and chicken (30.9%) represent the primary sources. This distribution strongly suggests that CC-443 strains possess a significant potential for foodborne transmission from poultry to humans potentially due to their enhanced stress tolerance. The ability of these strains to withstand various environmental stressors, including heat, may contribute to their survival during food processing and storage, thereby increasing their likelihood of causing human infections. However, further studies are still needed to investigate the potential threat of CC-443 in human infections.

Previous studies on the heat shock proteins of *C. jejuni* have revealed their transcriptomic and proteomic changes and binding of heat shock regulators to promoter regions (14–16). However, the specific function of the heat shock chaperones in thermotolerance in *C. jejuni* has not been thoroughly investigated. Therefore, it is fundamental to understand how chaperones function in *C. jejuni*. In this study, we evaluated the function of heat shock chaperones in the survival of *C. jejuni* under heat stress. Interestingly, among the knockout mutants of heat shock chaperones, only the Δ*clpB* mutant showed a significant difference in viability compared to WT under heat treatment (Fig. 2A). The *D*-values, which represent the time required to reduce the bacterial population by 90% at a specific temperature, also showed a considerable difference in the Δ*clpB* mutant, indicating that *clpB* is a crucial genetic element in the thermotolerance of *C. jejuni* (Table 1). Additionally, the Δ*hspR* mutant exhibited significantly elevated levels of survival compared to WT (Fig. S2) possibly due to the elevated expression of *clpB*. The *hspR*-complemented strain showed slightly decreased survival compared to WT (Fig. S2), suggesting that the overexpression of HspR may lead to increased repression of *clpB* (18, 45, 46). Still, while our results indicated a possible regulatory effect of HspR on ClpB, a definitive elucidation of this relationship required additional studies involving double mutant analyses. A comprehensive understanding of the regulatory network governing heat shock responses in *C. jejuni* is critical.

It is widely recognized that *E. coli* ClpB cooperatively functions to disaggregate protein aggregates by directly binding to DnaK (20); thus, previous studies have focused on identifying the binding sites of ClpB and DnaK (19, 35). However, our results using the BACTH system showed that ClpB and DnaK of *C. jejuni* do not bind to each other (Fig. 3). This functional independence between ClpB and DnaK in *C. jejuni* may explain the different survival patterns observed in these mutants under heat treatment; the Δ*clpB* mutant showed markedly lower survival than WT at 50°C, whereas the viability of the Δ*dnaK* mutant remained comparable to that of WT (Fig. 2A). Although previous screenings may not have detected a direct interaction (47), the absence of such evidence in *C. jejuni* supports our hypothesis that ClpB operates without the cooperative binding typically observed in other organisms like *E. coli*. Furthermore, our data showing the significant impact of ClpB on survival under heat stress, even in the absence of DnaK, reinforce the notion that *C. jejuni* has developed unique adaptations for thermotolerance. While the stand-alone disaggregase ClpG has been reported to confer thermotolerance in *Pseudomonas aeruginosa* (39), further studies on ClpB of *C. jejuni* are necessary to determine whether ClpB functions independently as a standalone disaggregase or cooperates with a chaperone protein other than DnaK. Our results demonstrate that protein aggregates are formed at significantly higher levels in the absence of *clpB* compared to the WT strain during heat treatment (Fig. 4), potentially resulting in a marked decrease in the survival of the Δ*clpB* mutant. Collectively, our results show the disaggregating role of ClpB in *C. jejuni* under heat treatment without direct association with DnaK. It is worth noting that a better understanding is needed about which step ClpB takes its role, whether it acts as a protectant in the prior step of aggregation formation or disaggregates protein aggregates formed after heat stress.

Our study reveals a phylogenetic association with thermotolerance among *C. jejuni* strains. Certain clonal complexes, specifically CC-443 and CC-607, exhibit higher heat tolerance, while others, such as CC-21, demonstrate increased heat sensitivity (Fig. 1C). This knowledge can lead to targeted approaches in managing *C. jejuni* contamination based on the prevalence of tolerant CCs. Notably, we observed two distinct amino acid substitutions in the NTD and NBD of ClpB in the thermotolerant CC-443 group (Fig. 5). We also examined whether any of the other thermotolerant strains carried the same allele or shared point mutations with the CC-443 group (Fig. 5; Fig. S3). Our analysis revealed that while they may possess varying levels of heat tolerance, the specific unique substitutions observed in the CC-443 group (A20V and A596T) are indeed distinctive (Fig. S3). These domains play crucial roles in substrate binding and disaggregation activity (48, 49). The NTD fluctuates rapidly and mediates allosteric interactions with the regulatory middle domain and NBDs, thereby influencing ClpB’s overall activity (50). Previous research has shown that mutations in conserved amino acids in the NTD can enhance substrate binding (48), suggesting that the observed substitutions in CC-443 may contribute to its enhanced thermotolerance. The two ATP-binding sites located in the NBD-1 and NBD-2 of ClpB exhibit allosteric interactions that are essential for its chaperone function, with mutations in these sites affecting cooperativity and oligomerization properties (51). Especially, NBD-2 is essential for ClpB function, supporting an unfolding/threading mechanism for disaggregation (49). Moreover, the sequence preservation of NBD-2 seems to be an important factor of the ClpB function, as the affinity of NBD-1 depends on nucleotide binding to NBD-2 (52). Collectively, our results demonstrate that amino acid substitutions in the NTD and NBD-2 of ClpB in the CC-443 group can potentially impact its disaggregation activity through a complex interplay between structural elements and protein function. These modifications may contribute to the enhanced thermotolerance observed in this clonal complex. Altogether, while the unique substitutions observed in the ClpB of the thermotolerant CC-443 strains—specifically the A20V substitution in the N-terminal domain and the A596T change in the second nucleotide-binding domain (NBD-2)—are of interest, further studies are required to elucidate their precise roles in influencing the thermotolerant phenotype. Therefore, our findings suggest a potential correlation between these substitutions and enhanced thermotolerance capabilities, and we acknowledge the necessity for more rigorous experimental validation.

Here, we have demonstrated the significant role of ClpB in aiding the survival of *C. jejuni* under heat stress. Moreover, our findings indicate that specific phylogenetic groups of *C. jejuni* are associated with increased thermotolerance. ClpB enhances the survival of *C. jejuni* by alleviating protein aggregates accumulated due to exposure to heat stress. Our study suggests that the observed differences in thermotolerance among various *C. jejuni* lineages, particularly between clonal complexes CC-443 and CC-21, have significant consequences for food processing practices. The thermotolerance of CC-443 strains to heat stress indicates that traditional heat treatment methods, such as scalding at 50°C, may not be sufficient to ensure the elimination of all *C. jejuni* strains in poultry products. Understanding the molecular mechanisms of the thermotolerance of *C. jejuni* is essential for the effective control of this common foodborne pathogen.

## Data Availability

All data in this article are either already available (sources in the reference list) or will be made available on request.

## SUPPLEMENTAL MATERIAL

Supplemental figures and tables spectrum.02293-24-s0001.docx Fig. S1 to S3, and Table S1 and S2.

ASM does not own the copyrights to Supplemental Material that may be linked to, or accessed through, an article. The authors have granted ASM a non-exclusive, world-wide license to publish the Supplemental Material files. Please contact the corresponding author directly for reuse.

## References

[1] Burnham PM, Hendrixson DR. 2018. Campylobacter jejuni: collective components promoting a successful enteric lifestyle. Nat Rev Microbiol 16:551–565. doi:10.1038/s41579-018-0037-9 29892020

[2] Kaakoush NO, Castaño-Rodríguez N, Mitchell HM, Man SM. 2015. Global epidemiology of Campylobacter infection. Clin Microbiol Rev 28:687–720. doi:10.1128/CMR.00006-15 26062576 PMC4462680

[3] European Food Safety Authority. 2010. Analysis of the baseline survey on the prevalence of Campylobacter in broiler batches and of Campylobacter and Salmonella on broiler carcasses, in the EU, 2008. EFS2 8. doi:10.2903/j.efsa.2010.1503 PMC13090778 42006083

[4] Golden CE, Rothrock MJ Jr, Mishra A. 2021. Mapping foodborne pathogen contamination throughout the conventional and alternative poultry supply chains. Poult Sci 100:101157. doi:10.1016/j.psj.2021.101157 34089937 PMC8182426

[5] Blaser MJ. 1997. Epidemiologic and clinical features of Campylobacter jejuni infections. J Infect Dis 176 Suppl 2:S103–S105. doi:10.1086/513780 9396691

[6] Blaser MJ, Engberg J. 2008. Clinical aspects of *Campylobacter jejuni* and *Campylobacter coli* infections, p 99–121. In Nachamkin In, Szymanski CM, Blaser MJ (ed), Campylobacter, 3rd ed. ASM Press, Washington, DC.

[7] Acheson D, Allos BM. 2001. Campylobacter jejuni infections: update on emerging issues and trends. Clin Infect Dis 32:1201–1206. doi:10.1086/319760 11283810

[8] Gölz G, Kittler S, Malakauskas M, Alter T. 2018. Survival of Campylobacter in the food chain and the environment. Curr Clin Micro Rpt 5:126–134. doi:10.1007/s40588-018-0092-z

[9] Baek KT, Vegge CS, Skórko-Glonek J, Brøndsted L. 2011. Different contributions of HtrA protease and chaperone activities to Campylobacter jejuni stress tolerance and physiology. Appl Environ Microbiol 77:57–66. doi:10.1128/AEM.01603-10 21075890 PMC3019702

[10] Murphy C, Carroll C, Jordan KN. 2006. Environmental survival mechanisms of the foodborne pathogen Campylobacter jejuni. J Appl Microbiol 100:623–632. doi:10.1111/j.1365-2672.2006.02903.x 16553716

[11] Park SF. 2002. The physiology of Campylobacter species and its relevance to their role as foodborne pathogens. Int J Food Microbiol 74:177–188. doi:10.1016/s0168-1605(01)00678-x 11981968

[12] Konkel ME, Kim BJ, Klena JD, Young CR, Ziprin R. 1998. Characterization of the thermal stress response of Campylobacter jejuni. Infect Immun 66:3666–3672. doi:10.1128/IAI.66.8.3666-3672.1998 9673247 PMC108400

[13] Stintzi A. 2003. Gene expression profile of Campylobacter jejuni in response to growth temperature variation. J Bacteriol 185:2009–2016. doi:10.1128/JB.185.6.2009-2016.2003 12618466 PMC150132

[14] Palombo M, Scarlato V, Roncarati D. 2020. Cooperative regulation of Campylobacter jejuni heat-shock genes by HspR and HrcA. Microorganisms 8:1161. doi:10.3390/microorganisms8081161 32751623 PMC7464140

[15] Versace G, Palombo M, Menon A, Scarlato V, Roncarati D. 2021. Feeling the heat: the Campylobacter jejuni HrcA transcriptional repressor is an intrinsic protein thermosensor. Biomolecules 11:1413. doi:10.3390/biom11101413 34680046 PMC8533110

[16] Holmes CW, Penn CW, Lund PA. 2010. The hrcA and hspR regulons of Campylobacter jejuni. Microbiology (Reading) 156:158–166. doi:10.1099/mic.0.031708-0 19850618

[17] Roncarati D, Danielli A, Scarlato V. 2014. The HrcA repressor is the thermosensor of the heat-shock regulatory circuit in the human pathogen Helicobacter pylori. Mol Microbiol 92:910–920. doi:10.1111/mmi.12600 24698217

[18] Servant P, Mazodier P. 2001. Negative regulation of the heat shock response in Streptomyces. Arch Microbiol 176:237–242. doi:10.1007/s002030100321 11685367

[19] Miot M, Reidy M, Doyle SM, Hoskins JR, Johnston DM, Genest O, Vitery M-C, Masison DC, Wickner S. 2011. Species-specific collaboration of heat shock proteins (Hsp) 70 and 100 in thermotolerance and protein disaggregation. Proc Natl Acad Sci USA 108:6915–6920. doi:10.1073/pnas.1102828108 21474779 PMC3084080

[20] Rosenzweig R, Moradi S, Zarrine-Afsar A, Glover JR, Kay LE. 2013. Unraveling the mechanism of protein disaggregation through a ClpB-DnaK interaction. Science 339:1080–1083. doi:10.1126/science.1233066 23393091

[21] Yang H, Li Y, Johnson MG. 2001. Survival and death of Salmonella Typhimurium and Campylobacter jejuni in processing water and on chicken skin during poultry scalding and chilling. J Food Prot 64:770–776. doi:10.4315/0362-028x-64.6.770 11403124

[22] Oh E, Chui L, Bae J, Li V, Ma A, Mutschall SK, Taboada EN, McMullen LM, Jeon B. 2018. Frequent implication of multistress-tolerant Campylobacter jejuni in human infections. Emerg Infect Dis 24:1037–1044. doi:10.3201/eid2406.171587 29774830 PMC6004869

[23] Oh E, Andrews KJ, McMullen LM, Jeon B. 2019. Tolerance to stress conditions associated with food safety in Campylobacter jejuni strains isolated from retail raw chicken. Sci Rep 9:11915. doi:10.1038/s41598-019-48373-0 31417115 PMC6695378

[24] Hur JI, Kim J, Kang MS, Kim HJ, Ryu S, Jeon B. 2024. Cold tolerance in Campylobacter jejuni and its impact on food safety. Food Res Int 175:113683. doi:10.1016/j.foodres.2023.113683 38129027

[25] Hur JI, Kim J, Ryu S, Jeon B. 2022. Phylogenetic association and genetic factors in cold stress tolerance in Campylobacter jejuni. Microbiol Spectr 10:e0268122. doi:10.1128/spectrum.02681-22 36314968 PMC9769813

[26] Bucca G, Hindle Z, Smith CP. 1997. Regulation of the dnaK operon of Streptomyces coelicolor A3(2) is governed by HspR, an autoregulatory repressor protein. J Bacteriol 179:5999–6004. doi:10.1128/jb.179.19.5999-6004.1997 9324243 PMC179499

[27] Kim J, Park H, Kim J, Kim JH, Jung JI, Cho S, Ryu S, Jeon B. 2019. Comparative analysis of aerotolerance, antibiotic resistance, and virulence gene prevalence in Campylobacter jejuni isolates from retail raw chicken and duck meat in South Korea. Microorganisms 7:433. doi:10.3390/microorganisms7100433 31658662 PMC6843641

[28] Parkhill J, Wren BW, Mungall K, Ketley JM, Churcher C, Basham D, Chillingworth T, Davies RM, Feltwell T, Holroyd S, Jagels K, Karlyshev AV, Moule S, Pallen MJ, Penn CW, Quail MA, Rajandream MA, Rutherford KM, van Vliet AH, Whitehead S, Barrell BG. 2000. The genome sequence of the food-borne pathogen Campylobacter jejuni reveals hypervariable sequences. Nature New Biol 403:665–668. doi:10.1038/35001088 10688204

[29] Cho E, Kim J, Hur JI, Ryu S, Jeon B. 2024. Pleiotropic cellular responses underlying antibiotic tolerance in Campylobacter jejuni Front Microbiol 15:1493849. doi:10.3389/fmicb.2024.1493849 39651349 PMC11622253

[30] Jeon B, Muraoka W, Scupham A, Zhang Q. 2009. Roles of lipooligosaccharide and capsular polysaccharide in antimicrobial resistance and natural transformation of Campylobacter jejuni. J Antimicrob Chemother 63:462–468. doi:10.1093/jac/dkn529 19147521 PMC2640156

[31] Karlyshev AV, Wren BW. 2005. Development and application of an insertional system for gene delivery and expression in Campylobacter jejuni. Appl Environ Microbiol 71:4004–4013. doi:10.1128/AEM.71.7.4004-4013.2005 16000815 PMC1169003

[32] de Jong AEI, van Asselt ED, Zwietering MH, Nauta MJ, de Jonge R. 2012. Extreme heat resistance of food borne pathogens Campylobacter jejuni, Escherichia coli and Salmonella typhimurium during cooking of chicken breast fillet during cooking. Int J Microbiol 2012:196841. doi:10.1155/2012/196841 22389647 PMC3282150

[33] Bang J, Choi M, Jeong H, Lee S, Kim Y, Ryu J-H, Kim H. 2017. Heat tolerances of Salmonella, Cronobacter sakazakii, and Pediococcus acidilactici inoculated into galactooligosaccharide. J Food Prot 80:1123–1127. doi:10.4315/0362-028X.JFP-16-456 28581334

[34] Chen SH, Fegan N, Kocharunchitt C, Bowman JP, Duffy LL. 2020. Effect of peracetic acid on Campylobacter in food matrices mimicking commercial poultry processing. Food Control 113:107185. doi:10.1016/j.foodcont.2020.107185

[35] Karimova G, Pidoux J, Ullmann A, Ladant D. 1998. A bacterial two-hybrid system based on a reconstituted signal transduction pathway. Proc Natl Acad Sci U S A 95:5752–5756. doi:10.1073/pnas.95.10.5752 9576956 PMC20451

[36] Doyle SM, Shastry S, Kravats AN, Shih Y-H, Miot M, Hoskins JR, Stan G, Wickner S. 2015. Interplay between E. coli DnaK, ClpB and GrpE during protein disaggregation. J Mol Biol 427:312–327. doi:10.1016/j.jmb.2014.10.013 25451597 PMC4297517

[37] Miller JH. 1972. Experiments in molecular genetics. Cold Spring Harbor Laboratory Press, Cold Spring Harbor, NY.

[38] Khodaparast L, Khodaparast L, Gallardo R, Louros NN, Michiels E, Ramakrishnan R, Ramakers M, Claes F, Young L, Shahrooei M, Wilkinson H, Desager M, Mengistu Tadesse W, Nilsson KPR, Hammarström P, Aertsen A, Carpentier S, Van Eldere J, Rousseau F, Schymkowitz J. 2018. Aggregating sequences that occur in many proteins constitute weak spots of bacterial proteostasis. Nat Commun 9:866. doi:10.1038/s41467-018-03131-0 29491361 PMC5830399

[39] Habib I, Uyttendaele M, De Zutter L. 2010. Survival of poultry-derived Campylobacter jejuni of multilocus sequence type clonal complexes 21 and 45 under freeze, chill, oxidative, acid and heat stresses. Food Microbiol 27:829–834. doi:10.1016/j.fm.2010.04.009 20630326

[40] Lee C, Franke KB, Kamal SM, Kim H, Lünsdorf H, Jäger J, Nimtz M, Trček J, Jänsch L, Bukau B, Mogk A, Römling U. 2018. Stand-alone ClpG disaggregase confers superior heat tolerance to bacteria. Proc Natl Acad Sci U S A 115:E273–E282. doi:10.1073/pnas.1712051115 29263094 PMC5777039

[41] Shen D, Coleman J, Chan E, Nicholson TP, Dai L, Sheppard PW, Patton WF. 2011. Novel cell- and tissue-based assays for detecting misfolded and aggregated protein accumulation within aggresomes and inclusion bodies. Cell Biochem Biophys 60:173–185. doi:10.1007/s12013-010-9138-4 21132543 PMC3112480

[42] Navarro S, Ventura S. 2014. Fluorescent dye ProteoStat to detect and discriminate intracellular amyloid-like aggregates in Escherichia coli. Biotechnol J 9:1259–1266. doi:10.1002/biot.201400291 25112199

[43] Tripathi P, Parijat P, Patel VK, Batra JK. 2018. The amino-terminal domain of Mycobacterium tuberculosis ClpB protein plays a crucial role in its substrate disaggregation activity. FEBS Open Bio 8:1669–1690. doi:10.1002/2211-5463.12509 PMC6168691 30338218

[44] Beinker P, Schlee S, Auvula R, Reinstein J. 2005. Biochemical coupling of the two nucleotide binding domains of ClpB: covalent linkage is not a prerequisite for chaperone activity. J Biol Chem 280:37965–37973. doi:10.1074/jbc.M506672200 16162497

[45] Werbeck ND, Kellner JN, Barends TRM, Reinstein J. 2009. Nucleotide binding and allosteric modulation of the second AAA+ domain of ClpB probed by transient kinetic studies. Biochemistry 48:7240–7250. doi:10.1021/bi900880c 19594134

[46] Roncarati D, Scarlato V. 2017. Regulation of heat-shock genes in bacteria: from signal sensing to gene expression output. FEMS Microbiol Rev 41:549–574. doi:10.1093/femsre/fux015 28402413

[47] Parrish JR, Yu J, Liu G, Hines JA, Chan JE, Mangiola BA, Zhang H, Pacifico S, Fotouhi F, DiRita VJ, Ideker T, Andrews P, Finley RL Jr. 2007. A proteome-wide protein interaction map for Campylobacter jejuni. Genome Biol 8:7. doi:10.1186/gb-2007-8-7-r130 PMC2323224 17615063

[48] Tanaka N, Tani Y, Tada T, Lee Y-F, Kanaori K, Kunugi S. 2006. The roles of conserved amino acids on substrate binding and conformational integrity of ClpB N-terminal domain. Biochemistry 45:8556–8561. doi:10.1021/bi0606804 16834329

[49] Lum R, Tkach JM, Vierling E, Glover JR. 2004. Evidence for an unfolding/threading mechanism for protein disaggregation by Saccharomyces cerevisiae Hsp104. J Biol Chem 279:29139–29146. doi:10.1074/jbc.M403777200 15128736

[50] Iljina M, Mazal H, Goloubinoff P, Riven I, Haran G. 2021. Entropic inhibition: how the activity of a AAA+ machine is modulated by its substrate-binding domain. ACS Chem Biol 16:775–785. doi:10.1021/acschembio.1c00156 33739813 PMC8056383

[51] Schlee S, Groemping Y, Herde P, Seidel R, Reinstein J. 2001. The chaperone function of ClpB from Thermus thermophilus depends on allosteric interactions of its two ATP-binding sites. J Mol Biol 306:889–899. doi:10.1006/jmbi.2001.4455 11243796

[52] Fernández-Higuero JÁ, Acebrón SP, Taneva SG, Del Castillo U, Moro F, Muga A. 2011. Allosteric communication between the nucleotide binding domains of caseinolytic peptidase B. J Biol Chem 286:25547–25555. doi:10.1074/jbc.M111.231365 21642426 PMC3138311

