# Erratum for Hur et al., “ClpB enhances thermotolerance in *Campylobacter jejuni* through protein disaggregation independent of DnaK”

**DOI:** 10.1128/spectrum.02898-25

**Published:** 2025-10-07

**Authors:** Jeong In Hur, Jinshil Kim, Sangryeol Ryu, Byeonghwa Jeon

## ERRATUM

Volume 13, no. 6, e02293-24, 2025, https://doi.org/10.1128/spectrum.02293-24. Page 1, grey box: The following note should be added. “Jeong In Hur and Jinshil Kim contributed equally to this work. The author order was determined on the basis of seniority.”

