# Supplementary material for: ClpB enhances thermotolerance in Campylobacter jejuni through protein disaggregation independent of DnaK
Source: Microbiol Spectr. 2025 Apr 30;13(6):e02293-24. doi: 10.1128/spectrum.02293-24 (PMC12131804; doi:10.1128/spectrum.02293-24)
Supplement: Supplemental figures and tables — Fig. S1 to S3, and Table S1 and S2. [file spectrum.02293-24-s0001.docx]

Viability at 50°C (Log_10_ CFU/mL)

R^2^ = 0.9877

R^2^ = 0.9600

R^2^ = 0.8576

WT

Δ*clpB*

*clpB* comp

Time (min)

2

3

4

5

6

7

10

9

8

10

20

30

40

50

60

**Fig. S1. Delta values from the survival model of *C. jejuni* at 50°C.** Comparison of delta values (time required for a 1 log CFU reduction) of *C. jejuni* strains exposed to heat stress. The black dot represents the reference strain, *C. jejuni* NCTC 11168. Orange and grey dots indicate D-values of the *ΔclpB* mutant and *clpB*-complemented strain under heat stress at 50°C, respectively. Dotted lines in corresponding colors represent the linear regression model for each strain. The experiment was performed in triplicate with consistent results. Error bars represent standard errors of the means. WT, *C. jejuni* NCTC 11168 wild type; *ΔclpB*, *ΔclpB* mutant; *clpB* comp, clpB-complemented strain.


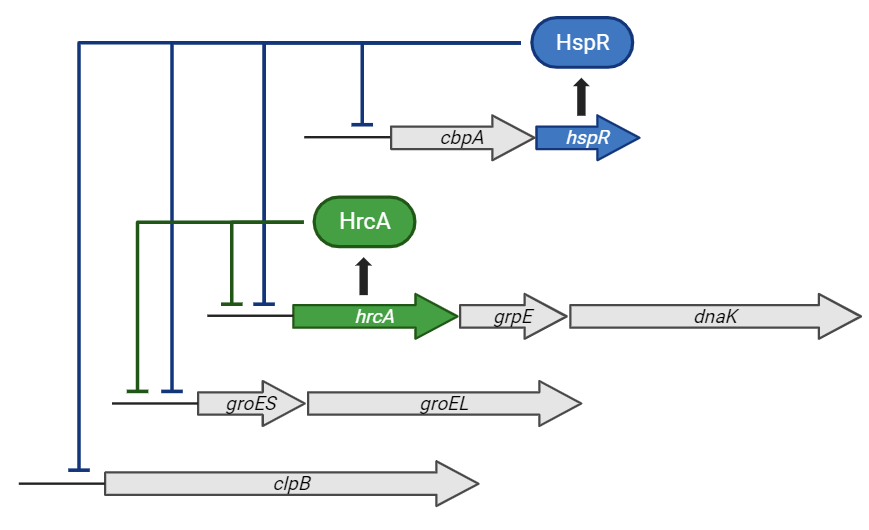


Viability (Log_10_ CFU/mL)

Time at 50°C (min)

WT

Δ*hspR*

*hspR* comp

Δ*hrcA*

*hrcA* comp

***

***

****

***

ns

ns

ns

ns

ns

ns

ns

ns

2

3

4

5

6

7

10

9

8

10

20

30

40

50

60

**A**

**B**

ns

ns

ns

ns

ns

ns

ns

ns

ns

ns

ns

ns

**Fig. S2. Role of heat shock regulators HrcA and HspR in *C. jejuni* under heat stress.** (A) Schematic representation of the negative regulation by heat shock regulators in *C. jejuni*, based on a previous study (1). (B) Survival of *C. jejuni* strains exposed to 50°C for 60 min. Asterisks indicate significant differences in viability between WT and other strains at the same sampling time. The dotted line represents the detection limit (200 CFU/mL). The experiment was performed in triplicate. Error bars indicate standard errors of the means. Statistical significance was determined using Student's *t*-test to compare viability between WT and the indicated mutant strains. . *, *P* < 0.05; **, *P* < 0.01; ns, non-significant. WT, *C. jejuni* NCTC 11168 wild type; Δ*hspR*, Δ*hspR* mutant; *hspR* comp, *hspR*-complemented strain; Δ*hrcA*, Δ*hrcA* mutant; *hrcA* comp, *hrcA*-complemented strain.


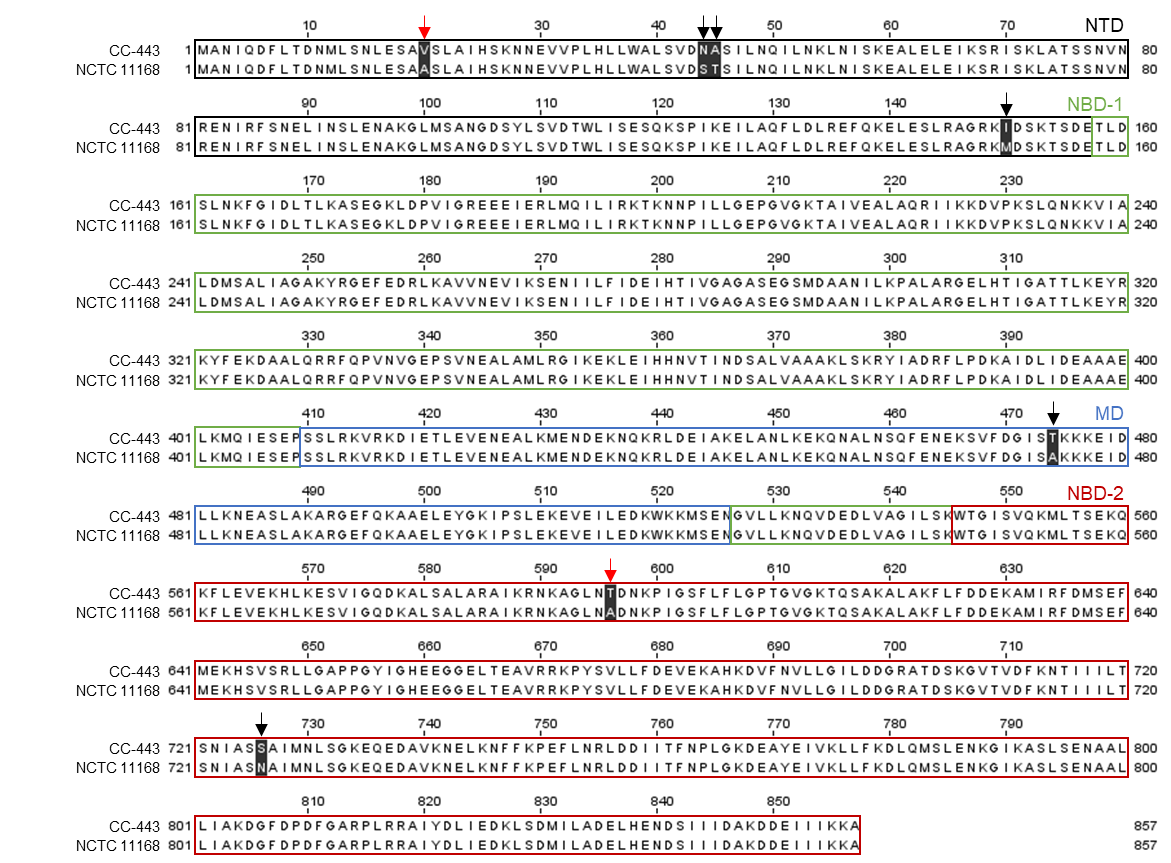


**Fig. S3. ClpB amino acid (AA) sequence alignment and domain prediction in *C. jejuni*.** Comparison of ClpB AA sequences and predicted domain organization between the CC-443 group and *C. jejuni* NCTC 11168. Seven highlighted regions with arrows indicate amino acid substitutions in ClpB of the CC-443 group compared to *C. jejuni* NCTC 11168. Red arrows highlight two amino acid substitutions that are fully conserved and unique to the CC-443 group. The domains of ClpB from *E. coli* MG1655 (857 amino acids) were used as a reference for domain prediction. NTD, N-terminal domain; NBD, nucleotide-binding domain; MD, middle domain.

**Table S1. Bacterial strains and plasmids used in this study**

| **Strain or plasmid** | **Genotype and relevant phenotype^a^** | **Reference or source** |
| --- | --- | --- |
| **Strains** | |  |
| *C. jejuni* | |  |
| NCTC 11168 | Wild type strain, a human isolate, reference for experiments | (2) |
| Δ*clpB* | Δ*clpB* mutant derived from NCTC 11168, *clpB*::*aphA-3* | (3) |
| *clpB* comp | *clpB* complementation using |  |
| Δ*dnaK* | Δ*dnaK* mutant derived from NCTC 11168, *dnaK*::*aphA-3* |  |
| *dnaK* comp | *dnaK* complementation |  |
| Δ*groESL* | Δ*groESL* mutant derived from NCTC 11168, *groESL*::*aphA-3* |  |
| *groESL* comp | *groESL* complementation |  |
| *E. coli* | | |
| DH5a | F− *endA1 glnV44 thi-1 recA1 relA1 gyrA96 deoR nupG* ϕ80d*lacZ*ΔM15 Δ(*lacZYA-argF*)*U169 hsdR17*(rK− mK+) λ− | Life Technologies |
| BTH101 | F− *cyaA-99 araD139 galE15 galK16 rpsL1 hsdR2 mcrA1 mcrB1* | Euromedex |
| **Plasmids** | | |
| pUC19 | Cloning vector used for suicide vector in *C. jejuni*; Amp^R^ | New England Biolabs |
| pMW10 | *E. coli*-*C. jejuni* shuttle vector; Km^R^ | (4) |
| pFMBcomCM | pUC19 derivatice carrying an rRNA gene cluster; Cm^R^ | (5) |
| pUT18C | Commercial vector encoding the T18 fragment for BACTH; Amp^R^ | (6) |
| pKT25 | Commercial vector encoding the T25 fragment for BACTH; Km^R^ |  |
| pUT18C-gene | Gene of interest cloned in XbaI and KpnI sites of pUT18C, resulting in N-terminal CyaA-T18-protein fusions (gene-T18) | This work |
| pKT25-gene | Gene of interest cloned in XbaI and KpnI sites of pKT25, resulting in N-terminal CyaA-T25-protein fusions (gene-T25) |  |

^a^Cm, chloramphenicol; Km, kanamycin

**Table S2. Primers used in this study**

| **Primer** | **Sequence (5'-3')** | **Reference** |
| --- | --- | --- |
| **Construction of *C. jejuni* mutants and complemented strains** | | |
| clpB-SalI-F | TTTGTCGACGCTTTATCTGCTGGTGTTCCTT | (3) |
| clpB-BamHI-R | AAAGGATCCGTATGCGTCAACCTGGAAGCT |  |
| clpB-inv-F | CGATATGATACTTGCTGATGAACTT |  |
| clpB-inv-R | GAATGTATAGCTAAAGATGCTGCA |  |
| clpB-comp-NotI-F | AAAGCGGCCGCGCAATCGTCTAAGTAGAACCATAG |  |
| clpB-comp-NotI-R | TTTGCGGCCGCGGAGAAAGTGCTTATTATACCAC |  |
| dnaK-SalI-F | AAAGTCGACGCTATAGAAGCAATGAAGAAAGAG |  |
| dnaK-BamHI-R | AAAGGATCCCCATCTCCCCTACTTGAACTG |  |
| dnaK-inv-F | GCGAAGTTTCTCATAAGTTAGCC |  |
| dnaK-inv-R | CGTTCATACACAGCAACACAAGA |  |
| dnaK-comp-NotI-F | AAAGCGGCCGCGAGTGGCTCTTATCAAAGATGAAA |  |
| dnaK-comp-NotI-R | TTTGCGGCCGCGTTTGCTTACACTAAAATGAATTGTCT |  |
| groESL-SalI-F | TTTGTCGACGCAATAGGGCTGTAATTATACATTC |  |
| groESL-BamHI-R | AAAGGATCCGCTAGCAATGAAGAAGTAAATACTA |  |
| groEL-inv-F | CGATCCTGTTAAAGTAGAAAGAGTA |  |
| groES-inv-R | CTAGAACACGCTTTCCTAAAGG |  |
| groESL-comp-NotI-F | AAAGCGGCCGCGCACAACAACAAAAGCTACAATG |  |
| groESL-comp-NotI-R | TTTGCGGCCGCGGAGGATTTGGTATAGGGCTTT |  |
| hrcA-SalI-F | TTAGTCGACCAGGCTTTAGAACCCCTTATG | This study |
| hrcA-BamHI-R | AAAGGATCCCCGTAAGCTAGAGCTGCTGA |  |
| hrcA-inv-F | GCTTAAGGTGGATGCGCAGT |  |
| hrcA-inv-R | GCGTATAGTCGAAGCAGGTAT |  |
| hrcA-comp-NotI-F | TGTGCGGCCGCACTTGACAAAATAACACTCAATGTTG |  |
| hrcA-comp-NotI-R | TGTGCGGCCGCGCTCAACATCTTCGATATTTTGCAA |  |
| hspR-SalI-F | ATTGTCGACGCTAGAAAATATCATCCTGATATCAA |  |
| hspR-BamHI-R | AAAGGATCCGCGCCACTACATCAAGTTGAAT |  |
| hspR-inv-F | CTAGTAAAGCCGTTGTTAAGCAT |  |
| hspR-inv-R | GCCTTAAGGTTTGTGGATGTATG |  |
| hspR-comp-XbaI-F | GACAGCAATCTAGAGCTTCACA |  |
| hspR-comp-XbaI-R | TATTCTAGACGGCTATATGAGTGATTTCGC |  |
| cat-phspho-F | TGCTCGGCGGTGTTCCTTT |  |
| cat-phspho-R | GCGCCCTTTAGTTCCTAAGG |  |
| Kan-F | GCGATGAAGTGCGTAAG | (7) |
| Kan-R | CGGCTCCGTCGATACTATG |  |
| **The BACTH assay** | | |
| clpB-XbaI-F | ATTCTAGACATGGCAAATATACAAGATTTTTTAACTGA | This study |
| clpB-KpnI-R | AATGGTACCTTAAGCTTTTTTGATGATTATTTCATCAT |  |
| dnaK-XbaI-F | ATTCTAGAGATGAGTAAAGTTATAGGTATAGATTTAG |  |
| dnaK-KpnI-R | TATGGTACCTTACTCAACTTCAGCGTCGATTA |  |
| EC-clpB-XbaI-F | ATTCTAGAGATGCGTCTGGATCGTCTTACTAA |  |
| EC-clpB-KpnI-R | TATGGTACCTTACTGGACGGCGACAATCCGGT |  |
| EC-dnaK-XbaI-F | AATCTAGACATGGGTAAAATAATTGGTATCGACC |  |
| EC-dnaK-KpnI-R | TATGGTACCTTATTTTTTGTCTTTGACTTCTTCAAATT |  |
| EC-clpB-conf-F1 | GAAGTGCCGGAAGGGTTGAAA |  |
| EC-clpB-conf-F2 | CAGACCATTAAAGCGGAACTG |  |
| EC-dnaK-conf-F1 | GTATCGCTGGTCTGGAAGTAAAA |  |
| EC-dnaK-conf-F2 | GTCTCTGGGTATCGAAACCAT |  |
| check-pUT18C-F | GGAAAAGCCTGTTCGACGATG | (8) |
| check-pKT25-F | GTCAAGGTGATCGGCAATGC |  |

*Underlining indicates the enzyme recognition sites.

**REFERENCES**

1. Versace G, Palombo M, Menon A, Scarlato V, Roncarati D. 2021. Feeling the heat: The *Campylobacter jejuni* HrcA transcriptional repressor is an intrinsic protein thermosensor. Biomolecules 11:1413.
2. Parkhill J, Wren B, Mungall K, Ketley J, Churcher C, Basham D, Chillingworth T, Davies R, Feltwell T, Holroyd S. 2000. The genome sequence of the food-borne pathogen Campylobacter jejuni reveals hypervariable sequences. Nature 403:665-668.
3. Cho E, Kim J, Hur JI, Ryu S, Jeon B. 2024. Pleiotropic cellular responses underlying antibiotic tolerance in Campylobacter jejuni. Front Microbiol https://doi.org/10.3389/fmicb.2024.1493849.
4. Wösten MM, Boeve M, Koot MG, van Nuenen AC, van der Zeijst BA. 1998. Identification of Campylobacter jejuni promoter sequences. J Bacteriol 180: 594–599.
5. Hwang S, Zhang Q, Ryu S, Jeon B. 2012. Transcriptional regulation of the CmeABC multidrug efflux pump and the KatA catalase by CosR in Campylobacter jejuni. J Bacteriol 194: 6883–6891.
6. Karimova G and Ladant D. 2005. A bacterial two-hybrid system based on a cyclic AMP signaling cascade, p 499–515. In Golemis E (ed), Protein-protein interactions. Cold Spring Harbor Laboratory Press, Cold Spring Harbor, NY.
7. Oh E, Jeon B. 2014. Role of alkyl hydroperoxide reductase (AhpC) in the biofilm formation of *Campylobacter jejuni*. PLoS One 9:e87312.
8. Zhang MX, Zheng KL, Tang AG, Hu XX, Guo XX, Wu C, Cheng YY. 2021. YgfY contributes to stress tolerance in *Shewanella oneidensis* neither as an antitoxin nor as a flavinylation factor of succinate dehydrogenase. Microorganisms 9:2316.
